# Community-based integrated care for patients with diabetes and depression (CIC-PDD): study protocol for a cluster randomized controlled trial

**DOI:** 10.1186/s13063-023-07561-0

**Published:** 2023-08-22

**Authors:** Yanshang Wang, Dan Guo, Ming Wang, Mingzheng Hu, Dawei Zhu, Qianqian Yu, Zhansheng Li, Xiaoyi Zhang, Ruoxi Ding, Miaomiao Zhao, Ping He

**Affiliations:** 1 School of Public Health, Peking University, Haidian District, 38 Xue Yuan Road, Beijing, 100191 China; 2 China Center for Health Development Studies, Peking University, Haidian District, 38 Xue Yuan Road, Beijing, 100191 China; 3 School of Management, Weifang Medical University, Weicheng District, 7166 Baotong Street, Weifang, 261053 Shandong China; 4 Health Commission of Weifang, 6396 Dongfeng East Street, Weifang, 261061 Shandong China; 5 Shanghai Mental Health Center, Shanghai Jiao Tong University School of Medicine, Xuhui District, 600 Wanping South Street, Shanghai, 200030 China; 6 Center for Mental Health Management, China Hospital Development Institute, Shanghai Jiao Tong University, Xuhui District, 600 Wanping South Street, Shanghai, 200030 China

**Keywords:** Multimorbidity, Integrated care, Diabetes, Depression, Implementation

## Abstract

**Background:**

Managing the multimorbidity of diabetes and depression remains a clinical challenge for patients and healthcare professionals due to the fragmented healthcare delivery system. To effectively cope with multimorbidity, there is an urgent need for the health system to transform into people-centered integrated care (PCIC) system globally. Therefore, this paper describes the protocol of community-based integrated care for patients with diabetes and depression (CIC-PDD) project, an integrated and shared-care intervention project.

**Methods/design:**

CIC-PDD project is conducted in two phases, namely “care model development” and “implementation and evaluation.” In the first phase, CIC-PDD model was designed and developed based on the four criteria of collaborative care model (CCM) and was subsequently adjusted to align with the context of China. The second phase entails a pragmatic, two-arm, cluster randomized controlled implementation trial, accompanied by parallel mixed-methods process evaluation and cost-effectiveness analysis.

**Discussion:**

We anticipate CIC-PDD project will facilitate the development and innovation of PCIC model and related theories worldwide, particularly in low- and middle-income countries (LMICs). In addition, CIC-PDD project will contribute to the exploration of primary health care (PHC) in addressing the multimorbidity of physical and mental health issues.

**Trial registration:**

ClinicalTrials.gov registration ChiCTR2200065608 (China Clinical Trials Registry https://www.chictr.org.cn). Registered on November 9, 2022.

**Supplementary Information:**

The online version contains supplementary material available at 10.1186/s13063-023-07561-0.

## Background

It is becoming increasingly apparent that multimorbidity has emerged as one of the greatest challenges confronting the health care system, both presently and in the coming decades [1]. Perhaps there is not a greater challenge than providing effective healthcare for patients with coexisting mental and physical multimorbidity, which is common, debilitating, and exacerbated by socioeconomic circumstances [2, 3].

### Diabetes and depression

Diabetes is the most common chronic metabolic disease, which represents a major burden on public health and healthcare systems [4]. Globally, there exist approximately 200 million individuals with diabetes in the world, and the number is expected to increase to 592 million by 2035 [5]. China has the highest number of individuals diagnosed with diabetes in the world [6]. A national representative survey conducted in China revealed a diabetes prevalence rate of 10.9%, with over 60% of cases remaining undiagnosed [7, 8].

Among all mental health problems, depression has the highest burden of disease. Over the past decade, depression has emerged as the second most prevalent cause of disability in China [9]. In the elderly, depression is common, particularly among those who have chronic conditions [10].

In diabetic patients, depression is the most prevalent mental health disorder [11, 12]. At least one-third of people with diabetes suffered from clinically relevant depressive disorders [13]. One systematic review across 48 studies conducted in low- and middle-income countries (LMICs) showed the concurrent prevalence of diabetes and depression was between 25 and 45%, with an average of 35.7%, which is significantly higher than that in high-income countries (HICs) [11]. In China, approximately 10% to 50% of type 2 diabetes (T2DM) patients suffer from depression [14], and the overall point prevalence is 28.9% [15].

### Burden and significance of co-morbid depression in patients with diabetes

It remains a clinical challenge for patients and healthcare professionals alike to manage the multimorbidity of diabetes and depression [16].

As a result of poor quality of life and diminished life expectancy, these patients experience a high degree of “illness burden”. This burden has a more profound impact on their well-being and health outcomes compared to a single diagnosis [16]. Consequently, depression in people with diabetes negatively affects glycemic control, increases the risks of microvascular and macrovascular complications [17], leads to increased diabetes-related distress [16], reduces quality of life [18], and increases mortality [19]. Similarly, they also suffer from “treatment burden” due to the necessity of visiting multiple specialist clinics. This situation is inconvenient for patients and inefficient for the health service [20, 21]. Furthermore, it can compromise patient self-care and decrease adherence to treatment [22].

### Rationale for integrated care

Providing integrated care (IC) is an efficient way to address the needs of people with diabetes and depression. A bold vision for IC was outlined by WHO in 2016, and it can be summarized as follows: “health services that are managed and delivered so that people receive a continuum of health promotion, disease prevention, diagnosis, treatment, disease-management, rehabilitation and palliative care services, coordinated across the different levels and sites of care within and beyond the health sector, and according to their needs throughout the life course” [23]. Within the broad category of integrated care, collaborative care model (CCM) is emerging as a promising approach. This model has demonstrated the potential to reduce healthcare costs, enhance physical and social functioning, and improve treatment adherence in the management of both mental illness and chronic physical conditions [24–27].

Our previous systematic review has demonstrated that IC is effective in reducing depression and improving quality of life for people with both depression and diabetes [28]. However, it is worth noting that the majority of studies implementing and evaluating the CCM have been conducted in HICs (USA: *n* = 10, Canada: *n* = 1, India: *n* = 1) [28]. There is significant uncertainty regarding whether these results are applicable to healthcare systems in LMICs [16, 28]. While experience from other countries can provide a valuable starting point, it is important to recognize that the implementation of the CCM involves complex and multifaceted interventions. Its practical implementation poses challenges on multiple levels, particularly in LMICs [29–31]. The cost-effectiveness of this model remains uncertain, as well as the optimal strategies for its implementation in routine practice [2].

### China context and the CIC-PDD study

There has been limited evidence of the effectiveness of IC in China. In recent years, China’s health care system is transitioning to a person-centered healthcare delivery system. In 2006, the Chinese Government issued guidelines on the development of primary health care (PHC), which strongly emphasize community-based management of chronic diseases [32]. This emphasis has stemmed from the increasing burden of non-communicable diseases (NCDs) [7]. Providing basic public health service package (BPHSP) to all people through government subsidies is one of the important initiatives [33]. This package includes various services, such as active screening, follow-up assessment, and health check-up services. In terms of mental health, management and patients with severe mental illness (e.g., schizophrenia and bipolar disorder) is included in the BPHSP.

Despite efforts to address the challenges faced by patients with diabetes and depression in China, the current healthcare system appears inadequate due to a lack of mental health resources and a fragmented delivery system. A report published in 2016, titled “deepening health reform in China,” proposed an upgrade to the healthcare system through the implementation of a tiered delivery system based on PCIC model [34]. This provides crucial theoretical framework for addressing the needs of patients with diabetes and depression.

Based upon the PCIC framework and CCM, we have developed and tested an integrated care model in China called Community-based Integrated Care for Patients with Diabetes and Depression (CIC-PDD). Our goal is to enhance the person-centeredness of the CIC-PDD model by developing self-management materials that are easily understandable in the local context. Additionally, we aim to identify strategies to overcome service discontinuities and enhance the mental health service capacity of primary healthcare providers (PCPs). Through these efforts, we seek to improve the overall care and outcomes for patients with both diabetes and depression in China.

## Methods/design

### Objectives

The study is designed to evaluate the real-world effectiveness of integrated, shared-care intervention project (CIC-PDD) compared to enhanced usual care (EUC) for patients with diabetes and depression in China.

### Study design

**Fig. 1:**
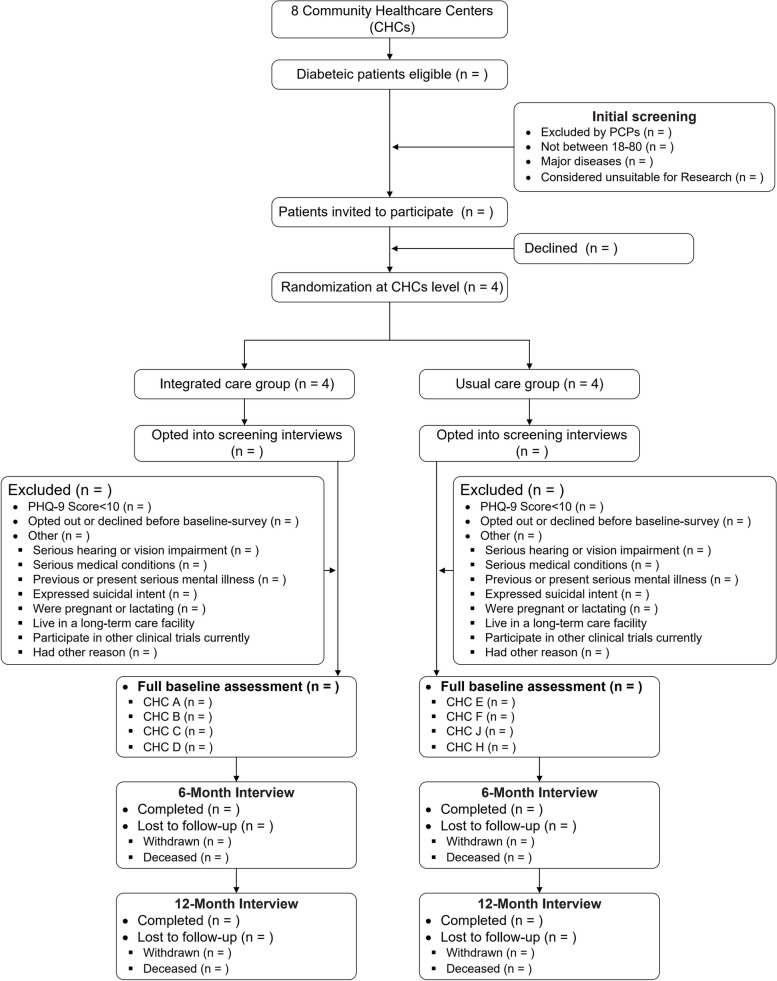
Design and flowchart of CIC-PDD

This is a pragmatic, two-arm, cluster randomized controlled implementation trial (Fig. 1), with parallel mixed-methods process evaluation and economic analysis of cost-effectiveness.

**Fig. 2:**
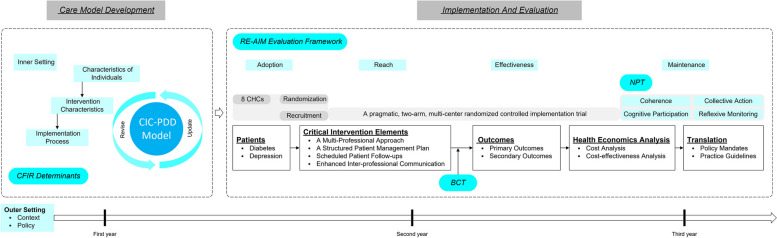
Theoretical frameworks informing CIC-PDD design and evaluation process

The design and implementation of the CIC-PDD project are guided by a wide range of theoretical and applied theories, as illustrated in Fig. 2. The following section provides a more detailed description of the design process.

#### RE-AIM

First, the design was shaped by Reach Effectiveness Adoption Implementation Maintenance (RE-AIM) and Consolidated Framework for Implementation Research (CFIR) that have been selected in light of the aims and context-relevant factors of CIC-PDD.

As a starting point, the RE-AIM evaluation framework provides a structured approach to assess the different stages of implementation and assists in selection of the most relevant and critical elements of the effectiveness and implementation outcome measures of CIC-PDD project.

#### CFIR

The CFIR will be utilized as a valuable tool to explore and complement the implementation components of RE-AIM. In order to identify and operationalize context-relevant barriers and facilitators for intervention adaptation [35], CFIR provides data to analyze pre-, during, and post-implementation. By leveraging the CFIR, we can identify relevant stakeholders and define constructs that are associated with the success of the implementation. Furthermore, we will utilize that to develop semi-structured interview topic guides and a policy analysis framework, and subsequently, to analyze the barriers and facilitators for implementation of CIC-PDD.

Two highly complementary theories, Behavior Change Theory (BCT) and Normalization Process Theory (NPT), will be used to explore and explain the causal mechanisms of change. Having obtained the results, it is essential to understand why the intervention work, and how to facilitate external scrutiny of their validity. Therefore, a holistic and multilayered framework is needed to grasp the changes in the behavior of individuals and organizations [36]. In this regard, Michie’s Theory is ideal and suitable [36] as it consolidates key components of the BCT, which will be used to identify the most pertinent and validated surveys. Additionally, it is also critical to address the implementation and execution of interventions in current healthcare settings. The conceptual framework is necessary to understand whether or not the intervention will be scalable and sustainable. Furthermore, we need to understand how the CIC-PDD would be adopted, implemented, practiced, and incorporated into current healthcare settings. NPT is ideal because it focuses on understanding the collective action and organizational behavior required to introduce complex and innovative interventions [37]. NPT will be used to gain a better understanding of the workability, feasibility, and sustainability of CIC-PDD model. It will assess the extent to which the model’s components can be assimilated and incorporated into routine practice. Within the overall evaluation framework, we can apply these theories to guide our quantitative and qualitative analysis (Fig. 2).

Figure 2 illustrates how these theoretical frameworks are used to inform the process for designing and evaluating the CIC-PDD project.

### Intervention design

#### CIC-PDD

**Fig. 3:**
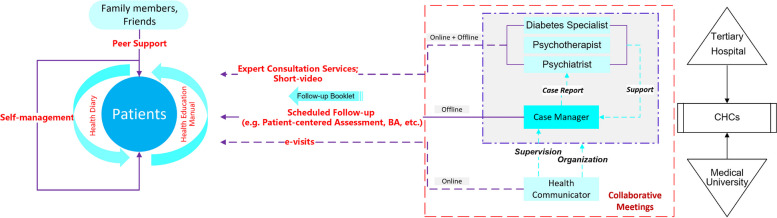
CIC-PDD model

The CIC-PDD model (Fig. 3) is developed based on four criteria of CCM established by Gunn [38] and is adjusted according to China health-care delivery system. Moreover, CIC-PDD model integrates new techniques, which highlight the importance of involvement of relatives and peer support in self-management [39].

The following introduces the CIC-PDD model components according to the four criteria of CCM:

1. A multi-professional approach

The multidisciplinary team consists of specialist team, case manager (CM), and health communicator. The functions of each role are as follows.

- Specialist team

The team consists of diabetes specialist, psychiatrist, and psychotherapist who are from tertiary hospitals. The main functions are as the follows: participate in collaborative meetings (online); provide expert consultation services for patients (offline); provide training and professional guidance to CMs; help CMs in making adjustments to the care plan; and regular video recording: provide ongoing training for CMs and support patient self-management.

- CM

A key component of CCM is the introduction of CM. The CM serves as a bridge between patients and professionals in primary and specialist care. They collaborate with patients to identify problems, set goals, develop action plans, and offer education and problem-solving skills to promote better patient self-care.

In the CIC-PDD project, the CMs will be acted by the PCPs. In a community healthcare center (CHC), the CMs consisted of general practitioners. In rural areas, village doctors serve as the CMs. The main functions are as the follows: proactive follow-up service to promote the management of patients for 18 sessions; measurement of blood glucose and PHQ-9 and fill out the “Follow-up Booklet”; support for the use of “Health Education Manual” and completion of “Health Diary”; attendance at team meetings to reporting the key patients’ conditions and making adjustments to the management plan.

- Health communicator

This role is a significant innovation of CIC-PDD model. We will recruit students who are in medical university to take on this role. The main functions are as follows: organizing team meeting and record contents; providing online health status assessments (e-visits): to verify the quality of the CM’s follow-up and to support self-management.

CMs can come from various professional backgrounds. The role of CM is typically performed by nurse in HICs [40]. Nurse-led CCM is feasible in settings in where nurses have already acquired extensive experience in managing chronic diseases. However, in China, nurses are not equipped in rural areas. In urban CHCs, nurses are mainly responsible for undertaking medical services and are rarely involved in public health. The follow-up service of our project is very structured and guided by guidelines and templates, demanding specialized medical-related consultation. Educating nurses to take on additional duties and adapt to new workflows may present more significant challenges [41, 42]. Additionally, preliminary interviews revealed the feasibility of assigning nurses to this role is relatively poor. Therefore, we set the PCPs as CMs, and we included the health communicator as the link between the CM and the specialist team. It can meet the needs of delivering IC in routine healthcare settings and also help improve the fidelity of the study (see below).

- (2) A structured patient management plan

All patients begin their IC by developing an individually structured treatment plan. This plan is developed based on the CIC-PDD manuals, following the stepped-care framework and complying with national guidelines.

The CIC-PDD intervention encompasses the following components:

- Patient-centered assessment and engagement: patients are usually first assessed in their own residential setting or CM’s workplace. The severity of depression, diabetes-related conditions and the relationship between depression and diabetes will be evaluated. It also encompasses an assessment of associated behavioral and social deficiencies. The evaluation results are described and recorded in “Follow-up Booklet.”
- Measurement and monitoring-based care: depression symptoms and HbA1c are measured at all sessions. These data serve as references for adjusting the care plan and helping care team make reasonable decisions.
- Behavioral activation (BA)-based care: The care plan focuses on behavioral activation (BA), which highlights the importance of engaging in pleasant or meaningful activities. This approach aims to help patients reschedule activities to reintroduce positive reinforcement, improve thoughts functioning and raise mood, and reduce the frequency of avoidant behaviors and unhealthy lifestyle. In collaboration with CM, patients will draw up goal-oriented behaviors likely to improve mental and physical health, and self-monitor and implement related activities according to schedules (“Health Diary”). Notably, BA is an effective and acceptable technique similar to cognitive behavioral therapy (CBT) [43]. In addition, BA is chosen because it employs a more perceptive therapeutic approach than CBT, which is easily delivered by PCPs after a short period of professional training [44].
- Technology-based care—an official WeChat official account called “Integrated Care -CIC-PDD” will be developed to address the accessibility of the care plan.

- (3) Scheduled patient follow-ups

All patients will receive proactive follow-up and case management from the assigned CM. Each patient will have a maximum of 18 sessions (two phases) over 1 year. The sessions can be conducted in-person or telephone contacts, depending on patients’ preference and condition.

- The first phase (high-intensity): 1–12 session

The frequency of follow-ups is every 2 weeks.

- The first session: CM needs to conduct a face-to-face contact with each patient lasting 30–60 min. Firstly, CM will briefly introduce the patient to the roles of all members of care team to build rapport. In addition, the CM will conduct a detailed Bio-psychosocial Semi-structured Assessment (BSA) based on the “Follow-up Booklet,” which review medical history, previous treatment measures for diabetes and depression and identifying key factors associated with low mood. The CM will also explore the patient’s experience of their behaviors, cognitive symptoms, and lifestyle. Together with the patient, the CM will develop a main problem statement. Additionally, the CM will introduce “Health Education Manual” and “Health Diary” as tools for recording daily task-lists and setting personal goals.
- The second session: In this session, CM will convey advice and recommendations from the specialist team to the patients. Together, the CM and the patient will develop a weekly task-list and set goals to be achieved. The CM will also introduce BA to engage the patient in the self-management process and develop simple strategies to cope with their problems. These strategies will incorporate interventions that address symptoms of depression and healthy lifestyle.
- For example, during this session, the CM may discuss self-management behaviors (e.g., increasing physical activity). The patients will also be educated about the relationship between diabetes and depression (mental health), emphasizing the importance of maintaining good mental health for effective blood glucose control. The CM will encourage patients to monitor mood and blood glucose on their own. Additionally, the patient’s adherence to prescribed medication will be assessed, and the CM will provide reliable and relevant information about the medication to support their understanding.
- The 3–13 session: During these sessions, CM will proactively maintain contact with the patients, primarily through face-to-face meetings. The CM will monitor the patients’ depressive symptoms (PHQ-9) and blood glucose. The CM will record the patient’s condition and any relevant information in the “Follow-up Booklet.” Moreover, BA is conducted to promote self-management and support compliance with care plan. For example, it helps individuals identify and implement a healthier lifestyle and encourages patients to engage in social interaction activities, such as participating in group activities and communicating frequently with friends and family.

If, during follow-up, it becomes apparent that symptoms are not improving, the CM and patients will collaboratively discuss options for further care plan.

- The second phase (low-intensity): 13–18 session

When the first phase is completed, the patients will have access to second phase regimens, but the frequency is once per month. Notably, the concept of “health maintenance” will be introduced during 15–18 sessions.

“After depressive symptoms and blood glucose levels reach the desired goal, it can and do relapse if not scientifically maintained, so prevention is important.”

In the final session, CM will review overall progress with patients and summarize the skills that patients can incorporate into routine practice.

Moreover, the health communicators also conduct e-visits (by telephone). The contents of e-visits include encouraging patients to take medication as prescribed, encouraging patients to take the initiative to contact with CM, and providing patients with some tips that can be used to improve their health, etc. According to the results of qualitative analysis in the pre-implementation period, e-visits are acceptable and patients can be engaged using this means of communication.

- (4) Enhanced inter-professional communication

To enhance inter-professional communication and facilitate management of patients, six 30 to 60-min collaborative meetings (online) will take place. It is not possible to establish a joint recording and communicating system between primary and secondary care system; however, online meetings can be held through existing electronic communication systems. The health communicator will organize collaborative meetings, which will focus on reviewing patients’ progress.

In the first meeting, the CM will report the health status of all managed patients based on results of the first session. The care team will review case together, and the specialist team will provide professional recommendations and guidance on the management plan. This includes individualized outreach, treatment intensification, and/or BA to support patients in achieving individualized goals.

For the 2–5 collaborative meetings, the CM only needs to present the changes in “key patients” whose depression and/or diabetes indicators are poorly controlled. The care team will review these patients’ progress with their main problem statement and goals, relevant health outcomes (e.g., PHQ-9, HbA1c), and medication. Afterwards, team will come together to discuss the next steps to be taken.

During the last collaborative meeting, changes in every patient’s condition need to be reported to the specialist team by the CM and health communicator. They will exchange and share their experiences and difficulties encountered in the CIC-PDD project and provide recommendations for the next step.

Figure 3 shows the potential components of CIC-PDD model.

- Additional elements

The additional elements of CIC-PDD include:

1. Intervention manual

The study team designed and developed detailed manuals. Different work manuals were used to identify their specific role and optimize process in care plan (these materials will not be published, available through the corresponding author). In addition, for patients’ manuals, the language was adapted to be easily understood. And to address the patient’s literacy and vision issues, we have added a lot of illustrations and cases to the manuals. We also encourage family members and CM to explain and disseminate the contents of these manuals to patients.

(2) WeChat official account (Integrated Care-CIC-PDD)

To promote patient self-management, as well as to record and monitor the project’s progress, the research team developed a WeChat public account (Integrated Care-CIC-PDD).) In CIC-PDD project, the specialist teams are all from the city while the majority of patients are from rural areas. Therefore, to optimize the management of patients, in CIC-PDD project, specialist teams are invited to film health short videos each month, which are disseminated to CMs and patients through “Integrated Care-CIC-PDD”. Additionally, “Integrated Care-CIC-PDD” is used to document research progress, share work experience, and present team assessment results, among other functions.

Based on our results of qualitative analysis, using fewer stigmatizing terms, rather than the psychiatric diagnoses, has been shown to reduce the stigma associated with mental disorders and enhance help-seeking. Therefore, in our interactions with patients, we will ask the care team to minimize the use of terms such as “depression” and “mental disorders” as much as possible, and instead use some easy-to-understand and acceptable terms such as mood.

In the implementation process, the duration and frequency of each follow-up will be flexibly adjusted according to the patient’s condition.

- EUC

In the control group, PCPs will be informed about patients’ depressive symptoms. Nevertheless, there is solid evidence that screening for depression does not result in improved treatment of depression [45]. Therefore, screening alone is unlikely to impact the quality of care available to patients, although patients in this group will still be eligible for anti-depressant medication and referrals for psychological treatment [46]. Notably, health management of diabetic patients is one service of BPHSC, and therefore the patients in control group will accept enhanced usual care (EUC).

Implementing intervention will not require alteration to usual care pathways and these will continue for both trial arms.

The trial participation is not expected to result in any harm to the participants, and there will be no provision for compensation for their involvement.

- Training

Care team in IC group will receive structured and targeted training, which includes both pre-intervention training and ongoing training during the intervention. Once the CHCs are randomly assigned, the CIC-PDD training team will provide offline training to the care team in IC group. Meanwhile, the care team received training on collaboration, as the successful implementation of care plan requires effective collaboration and cooperation within a multidisciplinary team. To familiarize team members with each other and recognize their roles, the study staff will organize on-site exercises, such as role-playing, case analysis, and content recording.

The study team compiles the training contents into the work manuals, which have been provided to the care team.

The details of the training are shown in Additional file 1: Table S1.

During the implementation phase, all CMs will receive ongoing support from specialist team and study staff. Our digital “Integrated Care-CIC-PDD” will continue to assist in enhancing the capabilities of CMs. This will be achieved through the provision of short videos on different topics, such as tips for improving sleep, diet recommendations, and correct usage of a blood glucose meter. Moreover, the study staff will provide video demonstrations, illustrating the proper use of the work manuals and sharing some typical cases as references.

### Patient and public involvement statement

In addition to the previously mentioned theoretical framework mentioned, we conducted focus groups with patients who have diabetes and depression to select key and effective outcomes and instruments for our study. The insights gained from these efforts were reviewed by study team, who then transformed these identified needs into research questions to be studied.

The concept of patient and public involvement also guided CIC-PDD model development. Since the core of IC is patient-centered care, before designing research plan and finalizing CIC-PDD model, we conduct multiple interviews with key stakeholders (especially patients) and engaged in multiple rounds of analysis, demonstration, and revision.

### Supervision and fidelity

CMs will receive ongoing supervision from health communicator and study staff. CMs need to keep and maintain notes for the patients using the “Follow-up Manual” during every session. This “Follow-up Booklet” reports how many sessions patients received, the delivery mode (in person or by telephone) in which they received intervention, PHQ-9 scores, blood glucose level, and what interventions patients accepted.

For each session, CMs are required to make audio recordings and take photos to document the process and capture important information. At the end of session, the CMs need to send the recorded materials to health communicator, who will review and evaluate them. A random selection of these materials will then be submitted to the study team for further assessment and analysis.

Second, the health communicator will also confirm whether the CMs conduct follow-up according to care plan from the patient’s perspective through regular e-visits.

Third, we will hold regular meetings with the directors of the CHCs, public health officials, and local health officials to ensure the fidelity of the CIC-PDD from the organizational perspective.

### Study setting

The study sites are 8 CHCs from two counties of Weifang, Shandong, a province in eastern China. There are several reasons for choosing Weifang as our study settings. First, this is a priority city for several initiatives to strengthen primary care, including the integrated chronic diseases care approach and the establishment of single disease group management. Additionally, Weifang is a national pilot city for the construction of social psychosocial service system designated by China National Department of Health, making it feasible to conduct the interventions of both mental and physical conditions. Furthermore, both selected counties have tertiary hospitals, which can meet the need for a multidisciplinary team of specialists.

Recruitment of CHCs ran from July 2021 to November 2021.

### Randomization and recruitment

#### Randomization

We randomized CHCs in each county on a 1:1 ratio, with an equal number of CHCs (*n* = 4) in each group. The allocation to the two arms was balanced based on size of the cluster, using the number of diabetic patients in each cluster.

Before implementation, allocation of clusters to each study arm was conducted by a statistician at the Peking University China Center for Health Development Studies. The statistician was not involved in implementation of the study.

To reduce the likelihood of bias caused by contamination and type II errors, cluster randomization was chosen. Patients within the clusters are allocated to the same group as their PCPs.

See Fig. 1 for an illustration of the randomization process.

#### Sample size and power calculations

The study is powered to detect between-arm differences in achieving the primary outcome at 12 months post-randomization. Based on previous literature [47], a sample size of 480 patients (240 per group) at 8 CHCs offered greater than 80% power (*α* = 0.05; intraclass correlation coefficient 0.03) to detect a 20% absolute risk difference between groups on the primary outcome. To account for a potential 10–15% loss to follow-up (e.g., leave the area, die or refuse to take part in the study at follow-up) and to provide greater robustness against type II error, 280 cases were included in each of the two groups in the actual study. According to our previous systematic review [28], previous study sample sizes ranged from 58 to 417. Therefore, we expect that our study will have the largest sample size and, consequently, the highest efficiency and statistical power.

#### Blinding

First, participants are not blinded to their treatment arms (as it is impossible to conceal the fact of collaboration or lack of it from the patients).

Second, it is not possible to blind care team since they are required to participate in additional training activities, as well as the introduction of specialist team and health communicator.

Third, all recruited outcome assessors are blinded to the status of the patient group and work independently of the intervention team and study team.

Finally, the groups are coded and anonymized to ensure that researchers are blinded throughout the entire analysis and writing process.

The design is open label with only outcome assessors being blinded so unblinding will not occur.

#### Patient screening and recruitment

Recruiting patients is often considered the most challenging part of conducting a cluster randomized controlled trial (RCT) [48]. Failing to recruit the desired number of participants can lead to costly and time-consuming extensions, as well as a decrease in statistical power.

To overcome these problems, we employ a two-phase approach to patient recruitment: initial screening and eligibility testing. The target population for this study consists of diabetic patients who are over 18 years old and exhibit depressive symptoms (indicated by a PHQ-9 score ≥ 10). Based on the estimated prevalence of depression among individuals with diabetes, we anticipate screening 4000 diabetes patients to achieve the desired sample size.

##### Initial screening

PCPs will contact diabetic patients and provide them with brief information about the CIC-PDD project, without mentioning terms related to depression. Then, PCPs will review existing electronic health records to identify preliminary qualified participants with diabetes. PCPs will receive compensation for initial screening.

##### Eligibility testing

Individuals who are found eligible after the initial screening will have the study details fully explained to them, and they will be invited to complete an eligibility test assessment through telephone or face-to-face surveys. During this phase, PCPs will conduct a detailed eligibility test questionnaire with potential participants.

To be eligible for the study, participants must meet the following inclusion criteria:

1. Age ≥ 18 and ≤ 85 years, and;
2. Confirmed diagnosis of diabetes, and;
3. PHQ-9 score ≥ 10, and;
4. Not serious hearing or vision impairment, able to complete telephone interviews, and;
5. Willingness to consent to randomization.

The PHQ-9 can be administered either in person or by telephone with similar results, making telephone assessments an effective method for screening for depression in PHC [49].

If any of the following conditions exist, individuals will be excluded from participation:

1. Have a serious medical condition and/or are in an advanced stage (e.g., heart disease, kidney failure, cancer, major organ failure), or;
2. Have been diagnosed with bipolar disorder or schizophrenia, are currently on antipsychotic medication or mood stabilizers, or require psychiatric treatment in a medical facility, or;
3. Have active suicidal thoughts and intent (item # 9 of the PHQ-9), or;
4. Are pregnant or lactating, or;
5. Live in a long-term care facility, or;
6. Currently participate in other clinical trials, or;
7. No fixed address or contact details, or;
8. PCPs have removed them from the practice diabetes database, or;
9. For other reasons, the PCPs considered it unsuitable to participate in this study.

The flow diagram of the recruitment and screening strategy is shown in Fig. 1.

#### Informed consent procedure

##### Consent to be screened for eligibility

During the initial screening, PCPs will provide patients with verbal informed consent for brief notification of the project and the need to review their health records. During the eligibility testing phase, the PCPs will obtain informed consent from patient in-person or by telephone. They will introduce the details of the CIC-PDD project, including the content of the intervention, group assignment, and follow-up assessment, while also conducting the recruitment process simultaneously.

##### Consent to participate in the trial

Within 1 month of the eligibility testing appointment, the study staff will contact PCPs and send the name list of patients who meet the inclusion criteria. Participants will be recruited into the study following contact of the PCPs and will be set up baseline assessments (offline) that will be conducted by blinded outcome assessors. Participants will provide written consent before completing the baseline questionnaire.

In the informed consent document, participants will be explicitly queried regarding their willingness to permit the utilization of their data in the event of their withdrawal from the trial. Additionally, participants will be asked to grant permission to the research team for sharing pertinent data with individuals from Peking University engaged in the research or relevant regulatory authorities, as appropriate. Importantly, this trial does not encompass the collection of biological specimens for storage.

### Data collection plan

Following the completion of the trial, all collected data will undergo analysis to develop an implementation blueprint. The outcomes, measures, and methods presented in this protocol have been evaluated for relevance and feasibility, and they were designed in collaboration with key stakeholders.

The data for the CIC-PDD project consists of interviewer-based data (face-to-face) and register data. Participants are interviewed face to face at baseline, and again after 6 and 12 months. Data collection through interviews will be conducted by outcome assessors trained in the specific instruments.

See Table 1 for schedule of enrolment, interventions, and assessments.

**Table 1:** Schedule of enrolment, interventions and assessments

Assessment level	Construct	Metric/Measure	Data Sources	Data type
***Reach***				
Individual	Characteristics of patients	▪ Eligibility criteria	Health records; Survey data	Quantitative
		▪ Demographic information	Survey data	Quantitative
		▪ Characteristics of patients who screened positive depression	Survey data	Quantitative
		▪ Characteristics of patients who were lost to follow-up	Document review (CIC-PDD project records)	Qualitative
Organization	Characteristics of CMs	▪ Characteristics of CMs who implement follow-ups according to the care plan		
	Contextual factors	▪ Ability to identify and manage targeted patient populations	Interview data (Study team and CMs)	Qualitative
		▪ Identified facilitators and barriers to recruitment	Interview data (CFIR interviews with CMs)	Qualitative
		▪ Identified recommendations for improvement	Interview data (CFIR interviews with care team)	Qualitative
***Effectiveness***				
Individual	Primary outcomes	▪ Primary outcomes: glycemic control (HbA1c); depression symptoms scores (SCL-20)	Health records; survey data	Quantitative
	Mental and physical health outcomes:	▪ Proportion of participants achieving significant reductions in individual outcomes: [50% ≥ reduction in SCL-20], [HbA1c ≥ 0.5% reduction]	Survey data	Quantitative
	Change in quality of life	▪ SF-12 ▪Diabetes Quality of Life	Survey data	Quantitative
	Experience of holistic patient-centered care	▪PACIC ▪Satisfaction with care	Interview data (interviews with patients); survey data	Qualitative & quantitative
	Person-centered care	▪PCCA	Survey data	Quantitative
***Adoption***				
Individual	Characteristics of care team	▪ Characteristics of CMs, health communicators and specialists participating in CIC-PDD project	Document review (CIC-PDD project records)	Qualitative & quantitative
Organization	Characteristics of CHCs	▪ Criteria for CHCs participation	Document review (CIC-PDD project records)	Qualitative & quantitative
		▪Features of participating CHCs	Document review (CHCs documents)	Qualitative & quantitative
		▪ Comparison of characteristics between intervention CHCs and control CHCs	Survey data	Quantitative
		▪ Description of usual care in control CHCs	Document review (CHCs documents)	Qualitative
		▪ CHCs’ Readiness: ORIC	Survey data	Quantitative
	Contextual factors	▪ Perception of extent to which CIC-PDD project has been adopted by CHCs and modified to fit their context	Interview data (CFIR interviews with CMs, the directors of the CHCs, public health officials)	Qualitative
		▪ Identified facilitators, barriers, and recommendations at organizational level	Interview data (CFIR interviews with care team)	Qualitative
***Implementation***				
Individual	Patients’ compliance	▪ The use of “Health Education Manual” and “Health Diary”	e-visits	Qualitative & quantitative
	Change in behaviors and perceptions:	▪ Self-management: SDSCA Burden of illness: MTBQ Burden of treatment: MMAS	Survey data	Quantitative
Organization	Development of CICPDD	▪ Development of materials (e.g., Work Manuals); data collection protocols and forms	Document review (CIC-PDD project records)	Qualitative
	Processes	▪ Screening, recruitment and outcomes assessments	Document review (CIC-PDD project records)	Qualitative
	Training	▪ Detailing training sessions, and training contents and materials provided; formal and written post-training tests; feedback of training	Document review (CIC-PDD project records); survey data	Qualitative & quantitative
	Service delivery	▪ Follow-up sessions, specialist consultations; time and frequency of service delivery; case review	Document review (CIC-PDD project records)	Qualitative
	Care team attitudes and perceptions	▪ Baseline clinician attitudes survey ▪ LIM ▪ Project Satisfaction ▪ Perceptions of Working in a Primary Health Care Team	Survey data	Quantitative
	Fidelity	▪ Completion of “Follow-up Booklet”	Document review (CIC-PDD project records)	Qualitative
		▪ CIC-PDD Adherence Questionnaire	Survey data	Quantitative
		▪ Extent to which project is delivered and received as intended	Interview data (CFIR interviews with care team and patients); document review (CIC-PDD project records)	Qualitative
	Contextual factors	▪ Identified facilitators and barriers to implementation	Interview data (CFIR interviews with CMs)	Qualitative
		▪ Identified recommendations for improvement	Interview data (CFIR interviews with health communicators)	Qualitative
	Economic Evaluation	▪ Reduction in costs; the relative cost-effectiveness	Survey data; health records	Quantitative
***Maintenance***				
Individual	Patients’ gains	▪ Knowledge and skills acquired	NPT interviews with patients; survey data	Qualitative & quantitative
		▪ Stability of effects of the CIC-PDD project on patient-level outcomes of effectiveness over time	Interviews with intervention group participants	Qualitative
Organization	PCPs’ gains	▪ Knowledge and skills acquired	NPT interviews with CMs	Qualitative
	Transform into practice	▪ Integration of aspects of the model into routine practice	NPT interviews with CMs	Qualitative

*CMs*, case managers; *CHCs*, community health centers; *CFIR*, Consolidated Framework for Implementation Research; *SCL-20*, The Symptom Checklist-20; *SF-12*, The12-Item Short Form Health Survey; *PACIC*, Patient Assessment of Chronic Illness Care; *PCCA*, Patient-Centered Care Assessment Tool; *ORIC*, Organizational Readiness for Implementing Change; *SDSCA*, Summary of Diabetes Self-Care Activities Questionnaire; *MTBQ*, Multimorbidity Treatment Burden Questionnaire; *MMAS*, Morisky Medication Adherence Scale; *LIM*, Level of Integration Measure; *NPT*, Normalization Process Theory

#### Training and inter-rater reliability

All outcome assessors will receive the necessary training in survey planning and relevant instruments, along with ongoing support and supervision. After completing the training, outcome assessors will be grouped, and each group will be assigned a leader (supervisor) who is a member of the study team.

The supervisor and trial manager will verify the accuracy and consistency of the data collected. During fieldwork, supervisor will monitor the interview process to ensure compliance with protocol specifications. Additionally, approximately 20% of all interviews will be randomly checked by supervisors for quality assurance.

#### Assessments

The CIC-PDD assessment approach and instruments incorporate both effectiveness and implementation outcome evaluations. The timing and content of the outcome assessments are delineated in Tables 1 and 2. The RE-AIM evaluation framework guides and informs the outcome assessments (Table 2). The primary effectiveness evaluations consist of patient-reported outcome measures, including assessments of the study’s primary and secondary outcomes (Table 1). Further detail regarding selected outcome assessments is described below.

**Table 2:**
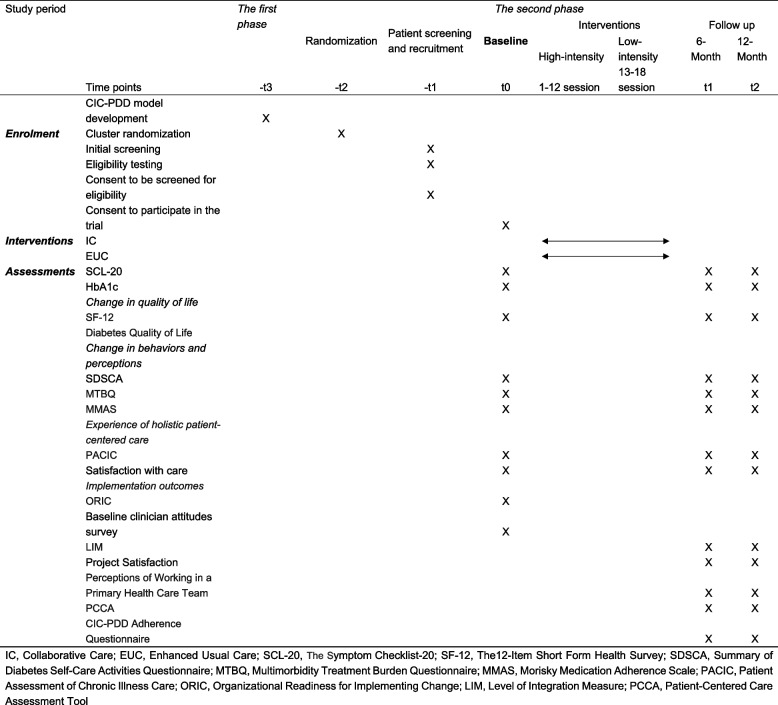
Key variables and data sources for assessment objectives

*IC*, Integrated Care; *EUC*, Enhanced Usual Care; *SCL-20*, The Symptom Checklist-20; *SF-12*, The12-Item Short Form Health Survey; *SDSCA*, Summary of Diabetes Self-Care Activities Questionnaire; *MTBQ*, Multimorbidity Treatment Burden Questionnaire; *MMAS*, Morisky Medication Adherence Scale; *PACIC*, Patient Assessment of Chronic Illness Care; *ORIC*, Organizational Readiness for Implementing Change; *LIM*, Level of Integration Measure; *PCCA*, Patient-Centered Care Assessment Tool

##### Primary outcome

The primary outcome in this trial is the change in depression symptoms scores (The Symptom Checklist-20, SCL-20) and glycemic control (HbA1c) from baseline to 6 and 12 months.

The use of the SCL-20 for outcome measurements is intended to minimize potential test–retest bias given the repeated use of the PHQ-9 for clinical care.

##### Secondary outcomes

To explore the causal mechanisms of CIC-PDD intervention, a number of secondary outcomes will be measured, using various instruments.

1. Mental and physical health outcomes
2. Proportion of participants achieving significant reductions in individual outcomes: reduced depressive symptoms [≥ 50% reduction in SCL-20], glycemic control [HbA1c ≥ 0.5% reduction].
3. Change in quality of life: general quality of life (The12-Item Short Form Health Survey, SF-12) [50] and disease-specific quality of life (Diabetes Quality of Life) [51].
4. Change in behaviors and perceptions
5. Self-management: Summary of Diabetes Self-Care Activities Questionnaire (SDSCA) [52]
6. Burden of illness: Multimorbidity Treatment Burden Questionnaire (MTBQ) [53]
7. Burden of treatment: Morisky Medication Adherence Scale (MMAS) [54]
8. Experience of patient-centered and coordinated care
9. Process measures: Patient Assessment of Chronic Illness Care (PACIC) [55]
10.  Satisfaction with care: overall satisfaction.
11.  Implementation outcomes

We have observed a lack of results on organizational factors at a system level and healthcare providers’ factors in previous studies, which are essential for reducing fragmented care and improving continuity and coordination [28]. Therefore, we will measure context characteristics, changes in health providers’ attitudes, and CIC-PDD adherence, which will help us gain a better understanding of our results.

- Community characteristics: Organizational Readiness for Implementing Change (ORIC) [56]
- Care team attitudes and perceptions: Baseline clinician attitudes survey, Level of Integration Measure (LIM) [57], Project Satisfaction [58], Perceptions of Working in a Primary Health Care Team [59]
- Collaborative care adherence: Patient-Centered Care Assessment Tool (PCCA) [60], CIC-PDD Adherence Questionnaire

##### Health care utilization

Using a service utilization questionnaire and electronic health records, we collect the number of clinic visits and hospital admissions, the use of resource use and related costs measures.

These outcomes, instruments, and data sources are summarized in Table 2.

There will be no biological specimens collected.

### Process evaluation

We will conduct a nested process evaluation in conjunction with the main analysis of quantitative results from the study. The process evaluation aims to explore the following objectives: (1) whether and how CIC-PDD project are implemented as intended (feasibility and acceptability), (2) to what extent this implementation aligns with the theory CIC-PDD project (consistency), and (3) which outcome has the greatest effectiveness and under what conditions or circumstances (sustainability).

In the process evaluation, the theories mentioned above (CFIR, RE-AIM, BCT, and NPT) are used to provide an overarching framework.

Furthermore, we will utilize a mixed-methods approach, combining qualitative and quantitative data throughout the study process. This approach includes administrative data, checklists, and logs completed at intervention sites, as well as surveys, semi-structured interviews, and focus group discussions.

### Health economics analysis

The objective of the economic analysis is to assess the relative cost-effectiveness of the CIC-PDD project.

The primary outcomes of the economic evaluation will be the incremental cost-effectiveness ratio (ICER) and the probability of cost-effectiveness, derived from the cost-effectiveness acceptability analysis. The measure of patient outcome will be quality-adjusted life years (QALYs) gained at the end of scheduled follow-up. To calculate health expenditures, costs will be classified as direct medical costs for health utilization, direct nonmedical costs (patient self-report), indirect costs (patient self-report), and costs of the intervention (e.g., costs of the training).

In the primary analyses, the perspective of health and social care service providers and patients will be taken into account. The cost-effectiveness acceptability curve (CEAC) will be derived from the analysis of covariance (ANCOVA) to estimate the probability of CIC-PDD project being more cost-effective than usual care across a range of ceiling thresholds.

We will conduct sensitivity analyses to examine the effects of varying discount rates, intervention costs, and effectiveness.

### Analysis

All analyses in CIC-PDD study will be performed based on an intention-to-treat statistical principle, adjusted for baseline characteristics [61]. Baseline, 6-month, and 12-month outcomes will be summarized by intervention arm and overall. The results will be presented as means (SD), medians (IQR), or numbers and proportions, as appropriate, with clustering taken into account.

Preliminary analysis will be conducted after the data have been cleaned to determine the pattern of missing baseline and follow-up data. We will conduct exploratory analyses to confirm expected distributions and assess the prevalence and patterns of missing data. Appropriate estimation techniques, such as multiple imputations, will be used to estimate missing data. Additionally, we will compare baseline characteristics between intervention and control group. The distribution of potential confounding factors will be taken into account as well as the participants who were able to complete baseline assessments and those who were unable to do so.

A multivariate regression model will be employed to estimate the effect of CIC-PDD on outcomes, accounting for potential confounding variables, including a random effect for CHCs and cluster size. Furthermore, we will explore the causal mechanism to gain a deeper understanding of how and why the results occur. Heterogeneity in treatment effects across sites and some baseline characteristics (e.g., sex, age, socioeconomic status) will be tested with interaction terms between these variables and the treatment status. To mitigate this effect of within-cluster correlation on estimator accuracy, we will cluster standard errors by CHCs. Moreover, sensitivity analysis will be carried out to check the robustness.

Additionally, we will conduct qualitative analysis to gain a better understanding of the implementation and policy processes, as well as to provide further insight into the results. All interviews and group discussions will be recorded and transcribed to capture the full information. Thematic analysis will be carried out on all qualitative data to identify themes. The theories outlined above will serve as the basis for developing interview topic guides and a coding framework.

Interim analyses and formal stopping rules are implemented to ensure the safety of participants and maintain integrity of the study results.

### Data management

Anonymity of participants will be protected through the removal of direct and indirect identifiers from the data during its analysis. We will retain data will for a period of at least 5 years after the project has been completed.

### The trial steering committee and data monitoring committee

The Trial Steering Committee (TSC) will be responsible for the independent supervision of the trial. Progress reports will be submitted by the research team to the TSC every 3 months. The TSC will closely monitor the study’s advancement and offer pertinent recommendations as needed.

Four external professionals who are not part of research team will serve as members of the data monitoring committee (DMC) for this trial. The committee will include a clinical trial expert, a biostatistician, a senior psychiatrist, and a senior diabetes specialist. The committee will be responsible for external oversight, monitoring the feasibility, data integrity, and safety of the study.

### Serious adverse events (SAEs) or adverse events (AEs)

Serious adverse events (SAEs) are not anticipated and we give a potential minor adverse events (AEs) list. The potential AEs that may occur during the course of the study include:

1. Mild hypoglycemia without the need for medical intervention.
2. Adverse effects of medications.
3. Weight gain.
4. Exacerbation of pre-existing conditions.
5. Mild to moderate retinopathy.
6. Common AEs in depression treatment include headache, dry mouth, insomnia, constipation, dizziness, fatigue, drowsiness, diarrhea, and sweating; most are mild to moderate in severity.

In the event of any SAES or AEs, regardless of their relation to the study intervention and irrespective of whether the intervention has been administered, immediate notification must be made within 24 h via telephone/fax to both the DMC and the principal investigator.

### Protocol amendments

When encountering deviations from the study protocol, the principal investigator will promptly report the situation. The method of timely reporting involves submitting a report to the Biomedical Ethics Committee of Peking University within 5 working days after the event occurs. Based on the feedback received, the principal investigator will assess and analyze the deviations from the study protocol, documenting this action in the progress report to identify potential trends that may indicate substantial issues. Furthermore, we will update the protocol in the clinical trial registry.

## Discussion

The CIC-PDD project aims to facilitate the development and innovation of PCIC model. Given the rising burden of NCDs, the high prevalence of concurrent diabetes and depression poses a significant and growing public health threat, especially in LMICs. Populations with diabetes and depression often struggle to adequate and reasonable care, despite the considerable illness and treatment burden that continues to increase. Worldwide, deficiencies in the availability, continuity, and quality of health services are considered key barriers to improving the health of this group, especially in LMCs. Urgent transformation of the health system into a PCIC system is needed. While CCM has been proven to be feasible and effective in some HICs, it remains unclear how integrated care models can be disseminated and implemented in routine care settings with limited resources, professional resistance, and competing priorities [28]. To our knowledge, CIC-PDD is the first study that is designed and carried out to explore effectiveness of CCM in China, and the first implementation study focused on patient with multimorbidity of physical and mental disorders. The results of CIC-PDD will contribute to the limited pool of knowledge about CCM and further the development and innovation of the concept and theory of integrated care globally. If the CIC-PDD project shows positive results, it will significantly improve future care for patients with multimorbidity in routine practice.

The CIC-PDD project will help to explore the potential of PHC in addressing mental health issues. A study found that only 0.5% of respondents with depressive disorders received adequate treatment in China, a number significantly lower in PHC context [62]. Treatment adequacy for major depressive disorder was merely 9.2%, even in specialist mental health services [32, 62]. The major reason behind this is the shortage of mental health resources, particularly in rural areas [63]. Therefore, there is an urgent need to close the treatment gap, especially for patients who have co-morbid chronic conditions. Empowering PCPs to deliver mental health services can help address the gaps in accessibility. However, studies have reported that PCPs are often inadequately trained, which hinders their ability to detect and manage common mental disorders effectively [62, 64]. Task-sharing has been recommended as a major strategy to address workforce shortages and close this gap, given the lack of mental health specialists in LMICs. Growing evidence shows that non-specialists can be trained to identify, diagnose, and treat patients suffering from mental health problems, leading to improved adherence and clinical outcomes [65–68]. Although the CIC-PDD project is designed for patients with diabetes and depression, we anticipate that the training of PCPs, the delivery of CC, and deployment of specialist teams will greatly enhance PCPs’ capacity to provide mental health service and improve access to and quality of mental health services for underserved population.

There will be several unintended and additional benefits derived from CIC-PDD, including (1) identification of unrecognized depressive symptoms among several thousand diabetic patients; (2) reducing PCPs’ stigma toward mental health disorders; and (3) facilitating the extension of mental and physical health services and the dissemination of advanced concepts (e.g., integrated care, CCM).

Additionally, it is anticipated that the CIC-PDD project has the potential to translate project results into policy. It will be the first RCT evaluating the real-world effectiveness of a task-shared collaborative team approach in China. Furthermore, by empowering and enhancing the skills of PCPs, the CIC-PDD project can lead to a lasting organizational change in the way patients with multimorbidity are managed in PHC. The study team has developed a stakeholder partnership with local health commission over the past decade, which will enable the results of CIC-PDD project to be translated into policy mandates and practice guidelines.

Inevitably, we anticipate that the implementation of the CIC-PDD project will face several challenges and limitations. One challenge is to provide PCPs with the necessary skills to deliver IC to patients in partnership with specialist team and health communicator, given that PCPs may have little mental health-related knowledge and are primarily engaged in clinical work. Another challenge is overcoming the complex interactions between mental and physical health system and establishing an efficient connection between the two.

The main limitation of this study is the fact that it is conducted in China, and the findings may not be immediately generalizable to other contexts of healthcare system. However, the CIC-PDD project aims to integrate interventions into existing healthcare delivery systems, which will provide insights into similar healthcare delivery structures in other regions.

### Trial status

The original version of the protocol (version 1.1 dated Sep 2, 2021) was approved by PU REB on October 12, 2021. Current version 1.2 (dated Sep 11, 2022) was approved by PU REB on October 11, 2022. The trial was registered on ClinicalTrials.gov on November 9, 2022. Recruitment commenced on December 1, 2022, and completed on January 20, 2023.

### Supplementary Information

**Additional file 1.**

**Additional file 2.**

## Data Availability

The datasets utilized for analysis in the present study, along with the statistical code, can be obtained from the corresponding author upon reasonable request. Additionally, the complete protocol is also accessible upon request from the corresponding author.

## Abbreviations

LMICs: Low- and middle-income countries
T2DM: Type 2 diabetes
CCM: Collaborative care model
IC: Integrated care
PCIC: People-centered integrated care
PHC: Primary health care
NCDs: Non-communicable diseases
BPHSP: Basic public health service package
CIC-PDD: Community-based integrated care for patients with diabetes and depression
PCPs: Primary health providers
EUC: Enhanced usual care
RE-AIM: Reach Effectiveness Adoption Implementation Maintenance
CFIR: Consolidated Framework for Implementation Research
BCT: Behavior Change Theory
NPT: Normalization Process Theory
CM: Case manager
CHC: Community health center
BA: Behavioral Activation
CBT: Cognitive Behavioral Therapy
BSA: Bio-psychosocial Semi-structured Assessment
RCT: Randomized controlled trials
SCL-20: The Symptom Checklist-20
SF-12: The12-Item Short Form Health Survey
SDSCA: Summary of Diabetes Self-Care Activities Questionnaire
MTBQ: Multimorbidity Treatment Burden Questionnaire
MMAS: Morisky Medication Adherence Scale
PACIC: Patient Assessment of Chronic Illness Care
ORIC: Organizational Readiness for Implementing Change
LIM: Level of Integration Measure
PCCA: Patient-Centered Care Assessment Tool
ICER: Incremental cost-effectiveness ratio
QALYs: Quality-adjusted life years
CEAC: Cost-effectiveness acceptability curve

## References

[1] Pearson-Stuttard J, Ezzati M, Gregg EW (2019). Multimorbidity-a defining challenge for health systems. Lancet Public Health.

[2] Gunn J. Designing care for people with mixed mental and physical multimorbidity. Bmj-Brit Med J. 2015;350. 10.1136/bmj.h712 25690275

[3] McLean G, Gunn J, Wyke S, Guthrie B, Watt GCM, Blane DN, Mercer SW. The influence of socioeconomic deprivation on multimorbidity at different ages: a cross-sectional study. Brit J Gen Pract. 2014;64(624):E440–7. 10.3399/bjgp14X680545 PMC4073730 24982497

[4] Wozniak L, Rees S, Soprovich A, Al Sayah F, Johnson ST, Majumdar SR, Johnson JA (2012). Applying the RE-AIM framework to the Alberta's Caring for Diabetes Project: a protocol for a comprehensive evaluation of primary care quality improvement interventions. BMJ Open.

[5] Pashaki MS, Mezel JA, Mokhtari Z, Gheshlagh RG, Hesabi PS, Nematifard T, Khaki S. The prevalence of comorbid depression in patients with diabetes: A meta-analysis of observational studies. Diabetes Metab Syndr. 2019;13(6):3113–9. 10.1016/j.dsx.2019.11.003 31790965

[6] Yang WY, Lu JM, Weng JP, Jia WP, Ji LN, Xiao JZ, Shan ZY, Liu J, Tian HM, Ji QH, et al. Prevalence of diabetes among men and women in china. N Engl J Med. 2010;362(12):1090–101. 10.1056/NEJMoa0908292 20335585

[7] Yip W, Fu H, Chen AT, Zhai T, Jian W, Xu R, Pan J, Hu M, Zhou Z, Chen Q (2019). 10 years of health-care reform in China: progress and gaps in Universal Health Coverage. Lancet.

[8] Wang L, Gao P, Zhang M, Huang Z, Zhang D, Deng Q, Li Y, Zhao Z, Qin X, Jin D (2017). Prevalence and **ethnic pattern of diabetes and prediabetes in China in 2013**. JAMA.

[9] Yang G, Wang Y, Zeng Y, Gao GF, Liang X, Zhou M, Wan X, Yu S, Jiang Y, Naghavi M (2013). Rapid health transition in China, 1990–2010: findings from the Global Burden of Disease Study 2010. Lancet.

[10] Overend K, Lewis H, Bailey D, Bosanquet K, Chew-Graham C, Ekers D, Gascoyne S, Hems D, Holmes J, Keding A (2014). CASPER plus (CollAborative care in Screen-Positive EldeRs with major depressive disorder): study protocol for a randomised controlled trial. Trials.

[11] Anderson RJ, Freedland KE, Clouse RE, Lustman PJ (2001). The prevalence of comorbid depression in adults with diabetes - **a meta-analysis**. Diabetes Care.

[12] Rapp SR, Parisi SA, Walsh DA (1988). Psychological dysfunction and physical health among elderly medical inpatients. J Consult Clin Psych.

[13] Lloyd CE, Pambianco G, Orchard TJ (2010). Does diabetes-related distress explain the presence of depressive symptoms and/or poor self-care in individuals with Type 1 diabetes?. Diabet Med.

[14] Liu HQ, Xu XY, Hall JJ, Wu XS, Zhang M (2016). Differences in depression between unknown diabetes and known diabetes: results from China health and retirement longitudinal study. Int Psychogeriatr.

[15] Liu XB, Dong C, Jiang H, Zhong DL, Li YX, Zhang HL, Zhang J, Fan J, Li J, Guan L, et al. Prevalence and risk factors of depression in Chinese patients with type 2 diabetes mellitus: a protocol of systematic review and meta-analysis. Syst Rev-London. 2021;10(1). 10.1186/s13643-021-01855-7 PMC8620640 34823606

[16] Petrak F, Baumeister H, Skinner TC, Brown A, Holt RIG (2015). Depression and diabetes: treatment and health-care delivery. Lancet Diabetes Endocrinol.

[17] de Groot M, Anderson R, Freedland KE, Clouse RE, Lustman PJ (2001). Association of depression and diabetes complications: **a meta-analysis**. Psychosom Med.

[18] Moussavi S, Chatterji S, Verdes E, Tandon A, Patel V, Ustun B (2007). Depression, chronic diseases, and decrements in health: results from the World Health Surveys. Lancet.

[19] Baumeister H, Hutter N, Bengel J, Harter M (2011). Quality of Life in medically ill persons with comorbid mental disorders: a systematic review and meta-analysis. Psychother Psychosom.

[20] Smith SM, Soubhi H, Fortin M, Hudon C, O'Dowd T. Managing patients with multimorbidity: systematic review of interventions in primary care and community settings. Bmj-Brit Med J. 2012;345. 10.1136/bmj.e5205 PMC3432635 22945950

[21] Stewart M (2001). Towards a global definition of patient centred care - The patient should be the judge of patient centred care. Brit Med J.

[22] Petersen I, Kemp CG, Rao D, Wagenaar BH, Sherr K, Grant M, Bachmann M, Barnabas RV, Mntambo N, Gigaba S, et al. Implementation and scale-up of integrated depression care in South Africa: an observational implementation research protocol. Psychiatr Serv. 2021:appips202000014. 10.1176/appi.ps.202000014 PMC8410621 33691487

[23] Organization WH (2016). Report on the public consultation to inform development of the Framework on integrated people-centred health services.

[24] Tully PJ, Turnbull DA, Beltrame J, Horowitz J, Cosh S, Baumeister H, Wittert GA (2015). Panic disorder and incident coronary heart disease: a systematic review and meta-regression in 1 131 612 persons and 58 111 cardiac events. Psychol Med.

[25] Rutledge T, Hogan BE (2002). A quantitative review of prospective evidence linking psychological factors with hypertension development. Psychosom Med.

[26] Fan ZX, Wu YY, Shen J, Ji T, Zhan RY (2013). Schizophrenia and the risk of cardiovascular diseases: a meta-analysis of thirteen cohort studies. J Psychiatr Res.

[27] Firth J, Siddiqi N, Koyanagi A, Siskind D, Rosenbaum S, Galletly C, Allan S, Caneo C, Carney R, Carvalho AF (2019). The Lancet Psychiatry Commission: a blueprint for protecting physical health in people with mental illness. Lancet Psychiat.

[28] Wang Y, Hu M, Zhu D, Ding R, He P (2022). Effectiveness of collaborative care for depression and HbA1c in patients with depression and diabetes: a systematic review and meta-analysis. Int J Integr Care.

[29] Davy C, Bleasel J, Liu HM, Tchan M, Ponniah S, Brown A. Effectiveness of chronic care models: opportunities for improving healthcare practice and health outcomes: a systematic review. Bmc Health Serv Res. 2015;15. 10.1186/s12913-015-0854-8 PMC4448852 25958128

[30] Lyon AR, Whitaker K, Richardson LP, French WP, McCauley E (2019). Collaborative **care to improve access and quality in school-based behavioral health**. J School Health.

[31] Kolko DJ, McGuier EA, Turchi R, Thompson E, Iyengar S, Smith SN, Hoagwood K, Liebrecht C, Bennett IM, Powell BJ (2022). Care team and practice-level implementation strategies to optimize pediatric collaborative care: study protocol for a cluster-randomized hybrid type III trial. Implement Sci.

[32] Chen SL, Conwell Y, He J, Lu NJ, Wu JY (2015). Depression care management for adults older than 60 years in primary care clinics in urban China: a cluster-randomised trial. Lancet Psychiat.

[33] Meng Q, Mills A, Wang L, Han Q. What can we learn from China’s health system reform? bmj 2019, 365. 10.1136/bmj.l2349 PMC6598719 31217222

[34] Wang X, Sun X, Birch S, Gong F, Valentijn P, Chen L, Zhang Y, Huang Y, Yang H (2018). People-centred integrated care in urban China. Bull World Health Organ.

[35] Schoenthaler A, De La Calle F, Soto A, Barrett D, Cruz J, Payano L, Rosado M, Adhikari S, Ogedegbe G, Rosal M (2021). Bridging the evidence-to-practice gap: a stepped-wedge cluster randomized controlled trial evaluating practice facilitation as a strategy to accelerate translation of a multi-level adherence intervention into safety net practices. Implementation Science Communications.

[36] Michie S, van Stralen MM, West R: The behaviour change wheel: a new method for characterising and designing behaviour change interventions. Implementation Science 2011, 6. 10.1186/1748-5908-6-42 PMC3096582 21513547

[37] May CR, Mair F, Finch T, MacFarlane A, Dowrick C, Treweek S, Rapley T, Ballini L, Ong BN, Rogers A (2009). Development of a theory of implementation and integration: normalization process theory. Implement Sci.

[38] Gunn WB, Blount A (2009). Primary **care mental health: a new frontier for psychology**. J Clin Psychol.

[39] Fadlon I, Nielsen TH (2019). Family **health behaviors**. Am Econ Rev.

[40] Morgan M, Dunbar J, Reddy P, Coates M, Leahy R. The TrueBlue study: Is practice nurse-led collaborative care effective in the management of depression for patients with heart disease or diabetes? BmcFamPract. 2009;10. 10.1186/1471-2296-10-46 PMC2714499 19545446

[41] McDonald R, Campbell S, Lester H (2009). Practice nurses and the effects of the new general practitioner contract in the English National Health Service: The extension of a professional project?. Soc Sci Med.

[42] Macdonald W, Rogers A, Blakeman T, Bower P (2008). Practice nurses and the facilitation of self-management in primary care. J Adv Nurs.

[43] Ekers D, Richards D, Gilbody S (2008). A meta-analysis of randomized trials of behavioural treatment of depression. Psychol Med.

[44] Ekers D, Richards D, McMillan D, Bland JM, Gilbody S (2011). Behavioural activation delivered by the non-specialist: phase II randomised controlled trial. Br J Psychiatry.

[45] Gilbody S, Sheldon T, House A (2008). Screening and case-finding instruments for depression: a meta-analysis. Can Med Assoc J.

[46] Coventry PA, Lovell K, Dickens C, Bower P, Chew-Graham C, Cherrington A, Garrett C, Gibbons CJ, Baguley C, Roughley K (2012). Collaborative Interventions for Circulation and Depression (COINCIDE): study protocol for a cluster randomized controlled trial of collaborative care for depression in people with diabetes and/or coronary heart disease. Trials.

[47] Cummings DM, Lutes LD, Littlewood K, Solar C, Carraway M, Kirian K, Patil S, Adams A, Ciszewski S, Edwards S (2019). Randomized trial of a tailored cognitive behavioral intervention in type 2 diabetes with comorbid depressive and/or regimen-related distress symptoms: 12-month outcomes from COMRADE. Diabetes Care.

[48] Patterson S, Mairs H, Borschmann R (2011). Successful recruitment to trials: a phased approach to opening gates and building bridges. BMC Med Res Methodol.

[49] van Bastelaar KMP, Pouwer F, Geelhoed-Duijvestijn PHLM, Tack CJ, Bazelmans E, Beekman AT, Heine RJ, Snoek FJ (2010). Diabetes-specific emotional distress mediates the association between depressive symptoms and glycaemic control in **type 1 and type 2 diabetes**. Diabetic Med.

[50] Ware JE, Kosinski M, Keller SD (1996). A 12-item short-form health survey - Construction of scales and preliminary tests of reliability and validity. Med Care.

[51] Grp DR (1988). Reliability and validity of a diabetes quality-of-life measure for the diabetes control and complications trial (Dcct). Diabetes Care.

[52] Toobert DJ, Glasgow RE (1994). Assessing diabetes self-management: the summary of diabetes self-care activities questionnaire. Handbook of psychology and diabetes: A guide to psychological measurement in diabetes research and practice.

[53] Duncan P, Murphy M, Man MS, Chaplin K, Gaunt D, Salisbury C. Development and validation of the Multimorbidity Treatment Burden Questionnaire (MTBQ). Bmj Open. 2018;8(4). 10.1136/bmjopen-2017-019413 PMC5900423 29654011

[54] Morisky DE, Green LW, Levine DM (1986). Concurrent and predictive-validity of a self-reported measure of medication adherence. Med Care.

[55] Glasgow RE, Wagner EH, Schaefer J, Mahoney LD, Reid RJ, Greene SA (2005). Development and validation of the patient assessment of chronic illness care (PACIC). Med Care.

[56] Shea CM, Jacobs SR, Esserman DA, Bruce K, Weiner BJ. Organizational readiness for implementing change: a psychometric assessment of a new measure. Implementation Science. 2014;9. 10.1186/1748-5908-9-7 PMC3904699 24410955

[57] Staab EM, Terras M, Dave P, Beckman N, Shah S, Vinci LM, Yohanna D, Laiteerapong N (2018). Measuring perceived level of integration during the process of primary care behavioral health implementation. Am J Med Qual.

[58] Hine JF, Grennan AQ, Menousek KM, Robertson G, Valleley RJ, Evans JH (2017). Physician satisfaction with integrated behavioral health in pediatric primary care: consistency across rural and urban settings. J Prim Care Communit.

[59] Dieleman SL, Farris KB, Feeny D, Johnson JA, Tsuyuki RT, Brilliant S (2004). Primary health care teams: team members' perceptions of the collaborative process. J Interprof Care.

[60] Sidani S, Collins L, Harbman P, MacMillan K, Reeves S, Hurlock-Chorostecki C, Donald F, Staples P, van Soeren M (2014). Development of a measure to assess healthcare providers' implementation of patient-centered care. Worldv Evid-Based Nu.

[61] Porta N, Bonet C, Cobo E (2007). Discordance between reported intention-to-treat and per protocol analyses. J Clin Epidemiol.

[62] Lu J, Xu X, Huang Y, Li T, Ma C, Xu G, Yin H, Xu X, Ma Y, Wang L (2021). Prevalence of depressive disorders and treatment in China: a cross-sectional epidemiological study. Lancet Psychiat.

[63] Liang D, Mays VM, Hwang WC (2018). Integrated mental health services in China: challenges and planning for the future. Health Policy Plann.

[64] Que JY, Lu L, Shi L: Development and challenges of mental health in China. Gen Psychiat 2019, 32(1). 10.1136/gpsych-2019-100053 PMC6551437 31179426

[65] Hong Z, Qu B, Wu XT, Yang TH, Zhang Q, Zhou D (2009). Economic burden of epilepsy in a developing country: a retrospective cost analysis in China. Epilepsia.

[66] Chatterjee S, Naik S, John S, Dabholkar H, Balaji M, Koschorke M, Varghese M, Thara R, Weiss HA, Williams P (2014). Effectiveness of a community-based intervention for people with schizophrenia and their caregivers in India (COPSI): a randomised controlled trial. Lancet.

[67] Nizamie SH, Akthar S, Banerjee I, Goyal N (2009). Health care delivery model in epilepsy to reduce treatment gap: World Health Organization study from a rural tribal population of India. Epilepsy Res.

[68] Patel V, Xiao SY, Chen HH, Hanna F, Jotheeswaran AT, Luo D, Parikh R, Sharma E, Usmani S, Yu Y (2016). The magnitude of and health system responses to the mental health treatment gap in adults in India and China. Lancet.

