# Correction: Community-based integrated care for patients with diabetes and depression (CIC-PDD): study protocol for a cluster randomized controlled trial

**DOI:** 10.1186/s13063-024-08144-3

**Published:** 2024-05-02

**Authors:** Yanshang Wang, Dan Guo, Ming Wang, Mingzheng Hu, Dawei Zhu, Qianqian Yu, Zhansheng Li, Xiaoyi Zhang, Ruoxi Ding, Miaomiao Zhao, Ping He

**Affiliations:** 1 School of Public Health, Peking University, Haidian District, 38 Xue Yuan Road, Beijing, 100191 China; 2 China Center for Health Development Studies, Peking University, Haidian District, 38 Xue Yuan Road, Beijing, 100191 China; 3 School of Management, Weifang Medical University, Weicheng District, 7166 Baotong Street, Weifang, 261053 Shandong China; 4 Health Commission of Weifang, 6396 Dongfeng East Street, Weifang, 261061 Shandong China; 5 Shanghai Mental Health Center, Shanghai Jiao Tong University School of Medicine, Xuhui District, 600 Wanping South Street, Shanghai, 200030 China; 6 Center for Mental Health Management, China Hospital Development Institute, Shanghai Jiao Tong University, Xuhui District, 600 Wanping South Street, Shanghai, 200030 China

**Correction** **:** **Trials 24, 550 (2023)**

**https://doi.org/10.1186/s13063-023-07561-0**

Following publication of the original article [1], we have been notified that a correction is needed in the Trial status section, as the recruitment dates for the study were postponed due to the COVID-19 pandemic's impact in China.

Incorrect: "Recruitment commenced on January 12, 2022, and has completed in May 2022."

Correct: "Recruitment commenced on December 1, 2022, and completed on January 20, 2023."

The original article has been corrected.
