# Supplementary material for: Community-based integrated care for patients with diabetes and depression (CIC-PDD): study protocol for a cluster randomized controlled trial
Source: Trials. 2023 Aug 22;24:550. doi: 10.1186/s13063-023-07561-0 (PMC10464429; doi:10.1186/s13063-023-07561-0)
Supplement: Supplementary file 1 — Additional file 1. [file 13063_2023_7561_MOESM1_ESM.docx]

Appendix table 1 The training content

| Schedule | Trainer | Contents | Aims | Participants | | |
| --- | --- | --- | --- | --- | --- | --- |
|  |  |  |  | CM | Health communicator | Specialist team |
| The first session (a.m.) | Government Officials | Project background | To understand the background of 3CPDD project and the cooperation between the study team and the local government. | √ | √ | √ |
| The second session (a.m.) | Study Staff | Overview of the 3CPDD project | To understand the effects of CC on patients with diabetes and depression and the purpose of the 3CPDD project; to learn the use of related booklet/manuals. | √ | √ | √ |
| The third session (a.m.) | Psychotherapist | Depression management guidelines | To master the key points of depression management and the implementation of BA. | √ | √ |  |
| The first session (p.m.) | Endocrinologist | Diabetes management guidelines | To master the key points of diabetes management, and to learn the strategies to improve patients' medication and treatment compliance. | √ | √ |  |
| The second session (p.m.) | - | Exercises | To familiarize team members with each other and recognized their roles through some steps such as role-playing, case analysis and content recording. | √ | √ | √ |
|  |  | Post-training tests | To check the care team's understanding of 3CPDD project and their own roles. | √ | √ | √ |
| CC, Collaborative Care; BA, Behavioral Activation | | | | | | |
